# Exploring the genetic landscape of the Copper Homeostasis and Silver Resistance Island (CHASRI) in *Salmonella enterica*

**DOI:** 10.1371/journal.pone.0334908

**Published:** 2025-11-03

**Authors:** Julie Haendiges, Eric W. Brown, Christina Ferreira, Maria Hoffmann, Elizabeth Reed, Jie Zheng, Rohan Tikekar

**Affiliations:** 1 Human Foods Program, Food and Drug Administration, College Park, Maryland, United States of America; 2 Department of Nutrition and Food Science, University of Maryland, College Park, Maryland, United States of America; Islamic Azad University, IRAN, ISLAMIC REPUBLIC OF

## Abstract

Copper is essential for all living organisms, but becomes toxic when present in excess. Biological systems have evolved mechanisms to keep organisms in copper homeostasis. Studies have shown that *Salmonella* can acquire mobile genetic elements that provide enhanced tolerance to stressed environments, such as the Copper Homeostasis and Silver Resistance Island (CHASRI), which has become more prevalent in certain serovars that are exposed to higher levels of copper. In this study, whole genome sequence data available from NCBI Pathogen Detection was used to determine the incidence of the CHASRI is in *Salmonella enterica* isolates. The results show that the CHASRI is present in a wider range of serovars than previously known and can be found in isolates from different food sources, including nuts, spices, and produce. *Salmonella* Genomic Island-4 (SGI-4) was previously described as the primary mobile element through which the CHASRI was transferred to *Salmonella*; however, results from this comparative study of closed reference genomes identified additional integrations of the CHASRI as both a singular mobile element and as a component of an SGI-4 variant.

## Introduction

Copper is an essential micronutrient for bacteria; however, excessive copper is cytotoxic and must be controlled by different cellular copper pathways [1]. The majority of *Salmonella enterica* strains contain the Cue and Gol regulons to assist with copper homeostasis; however, these regulons are only effective in aerobic environments due to their mechanism of inactivation [1,2]. Alternatively, bacterial pathways that control copper toxicity in anaerobic environments are uncommon but would be beneficial for facultative anaerobes [1]. High levels of copper present in different environments have led to the integration of additional copper tolerance systems in bacteria [3,4].

The Copper Homeostasis and Silver Resistance Island (CHASRI) is one such system and can be found in *Enterobacteriaceae*. The CHASRI contains a copper homeostasis system *pco* (*pcoABCDRSE*), a heavy metal export system *sil* (*silEP*), and a copper-sensing efflux system *cus* (*cusABFCRS*), and has been identified on both plasmids and chromosomes [4]. The *pco* system was first identified on a plasmid, while the *cus* system was first identified in the chromosomal genome of two *Escherichia coli* strains [5,6]. The combination of these two operons provides protection in both aerobic and anaerobic conditions and during transitions between these states.

*Salmonella* Genomic Island-4 (SGI-4) was identified in I 4, [5],12:i:- and contains the CHASRI as well as an arsenical tolerance operon (*arsRDABC).* Previous studies have examined the presence of SGI-4 in *S.* I 4, [5],12:i:- strains in Europe and Japan [7,8]. Researchers hypothesized that copper in livestock feed may have driven the selection pressure for the *S.* I 4, [5],12:i:- clone [8]. Additionally, SGI-4 acquisition provides increased copper tolerance in anaerobic environments, which may aide growth in the intestinal tract of animals supplemented with heavy metals.

In addition to *S.* I 4, [5],12:i:-, the CHASRI is also associated with certain sequence types (ST) ST316 and ST14, which have been linked to previous outbreaks [9,10]. Additionally, it has been previously documented certain serovars (Typhimurium and Montevideo) and in isolates from certain sources (swine and poultry) [7,9]; however, the overall prevalence of this mobile element in *Salmonella* isolates is not fully defined.

Previously, we identified the CHASRI in a study of *S.* Senftenberg and Montevideo from contaminated pistachios and hypothesized that this genomic cassette may be identified in other *Salmonella* isolates associated with agriculture [7,10,11]. The purpose of this study was to address two key questions: 1) How is the CHASRI distributed across *Salmonella* genomes? and 2) What variations of the CHASRI are present among published *S. enterica* reference genomes? Answering these questions may provide insights into the role of the CHASRI in bacterial adaptation.

## Materials and methods

### Identification of *Salmonella* isolates containing the CHASRI found in NCBI Pathogen Detection

The source of the data for analysis was the *Salmonella* database within the NCBI Pathogen Detection (NCBI PD) browser (https://www.ncbi.nlm.nih.gov/pathogens/). This database provides metadata for the isolates as well as stress genotypes and computed serovars for all submitted isolates through the integration of AMRFinderPlus [12] and SeqSero2 [13]. NCBI PD uses the nomenclature *sil* for the *cus* operon based on the reference gene catalog; although the names differ, the sequences are identical.

The database was accessed in August 2024 by querying the entire Salmonella database and using filtering options to identify isolates with any of the following CHASRI genes: *silA, silB, silC, silE, silF, silP, silR, silS, pcoA, pcoB, pcoC, pcoD, pcoE*, *pcoR,* and *pcoS*. Metadata for the resulting isolates were exported for further analysis. Additional manual filtering was performed to remove any isolate that was missing an isolation source, had a blank “computed serotype” field, or had fewer than 80% of the queried genes. The cutoff of 80% was determined by looking at the number of hits of each individual genes of interest. Two genes (*pcoB* and *pcoE*) were not identified in most of the queried isolates. This could be due to assembly or library preparation biases.

### Standardization of isolation source metadata

The list of isolates was separated into two groups based on the NCBI “Isolation type” field (clinical, environmental/other). The “Isolation source” field for submission is free text and not standardized among submitting labs; therefore, standardized isolation sources were essential for comparison. This was achieved by utilizing the LexMapr program (https://github.com/cidgoh/LexMapr/tree/master/lexmapr) and the IFSAC hierarchical isolation source categorization scheme [14]. The output from LexMapr was then used to group the isolates into 25 categories. The 25 categories were: animal feed, beef, chicken, dairy, eggs, environmental, environmental-facility/production, environmental-farm, environmental-retail, environmental-slaughterhouse, environmental-soil, environmental-water, fish, fruit, grains/beans, herbs/spices, multi-ingredient food, nuts/seeds, other animal, other poultry, pork, shellfish, turkey, vegetable, and clinical. The livestock groupings (beef, chicken, pork, turkey) were kept broad and include no distinction between the animal or meat isolation sources. Isolates with a generic isolation source of “environmental” or “environment” that provided no other information were characterized as “environmental”.

Overall incidence rate of the CHASRI was calculated for the top serovars in the dataset as well as any additional serovars that are the most abundant in PD. The filtering options were used to first filter based on “computed serotype” and then the CHASRI genes. The total number of isolates and positive isolates were then used to calculate 95% confidence intervals using the Clopper-Pearson method and adjusted for multiplicity by the Bonferroni method. All calculations were performed using R Core Team (version 4.3.1)

### *In-silico* serotyping

The computed serotypes obtained from NCBI PD were verified using SeqSero2 by initially running the allele mode on the assemblies. A secondary analysis was performed on the raw reads if there were issues identifying a named serovar. Manual checks of the SNP clusters were performed for any isolates missing the SRA accession or if there were serotype discrepancies.

### Comparative sequence analysis of CHASRI sequences from reference genomes

The previous analysis was based on short read data; therefore, to elucidate how the CHASRI is being integrated and to identify any genomic variations of the CHASRI, closed reference sequences were analyzed. Reference genomes of *S. enterica* that contain the CHASRI were identified in NCBI, and the GenBank files were downloaded. The location of the CHASRI (chromosome vs. plasmid) was determined by the metadata associated with the sequence. In instances where the CHASRI could be found on both the chromosome and a plasmid in the same strain, both GenBank files were downloaded and included in the analysis.

In addition to the *Salmonella* isolates, CHASRI sequences used in a previous study (*Citrobacter freundii* (NZ_LS992183), *Escherichia coli* (KU248945), *Serratia marcescens* (NC_005211), and *Enterobacter cloacae* (NC_014107)) were downloaded and added to the analysis [4,15]. The nucleotide sequences of the entire CHASRI (all genes and intergenic regions) were aligned using MUSCLE [16], and the resulting alignment was used to produce the minimum spanning tree with RAxML [17]. The RAxML analysis was performed using default values, the GTRCAT nucleotide substitution model and 500 bootstrap replicates.

Determination of the presence of SGI-4 or the SGI-4 variant was performed using the same techniques by Li et al. with this dataset. Briefly, a BLAST query against the SGI-4 reference (MN730129.1) and the SGI-4 variant (OK209934.1) with 85% coverage and 70% identity cutoffs was performed [15]. PlasmidFinder was used to identify plasmid replicon sequences in those GenBank files where the CHASRI was located on a plasmid [18]. A subset of isolates was compared using the program clinker with the default settings to visualize the gene clusters found in SGI-4, SGI-4 variant, and the CHASRI mobile element [19].

## Results and discussion

### Distribution of the CHASRI in *S. enterica* from isolates on Pathogen Detection

The NCBI Pathogen Detection database is the largest public database of isolate sequences available. The *Salmonella* database contained 653,488 isolates (August 2024) at the time of analysis. However, isolates uploaded to the NCBI Pathogen Detection database do not represent a random sample of *Salmonella* present in all environments due to the fact that many isolates are collected as part of a routine sampling plan or during outbreak investigations. This may inflate the apparent frequency of CHASRI in lineages that are over-sampled while underestimating its presence in less studied reservoirs. Many genomes originate from certain regions or specific outbreak investigations, which also limits the generalizability of observed CHASRI patterns to all *Salmonella* populations. In addition, draft genomes with incomplete assemblies may miss or fragment CHASRI elements, leading to false negative. Genomes are more likely to be submitted if they are linked to notable phenotypes, which can skew CHASRI prevalence estimates as well. Therefore, the results describe CHASRI distribution in the set of available genomes rather than across all *Salmonella* globally. Findings would be framed as a baseline picture, not definitive prevalence estimate. The query performed in Pathogen Detection resulted in a total of 61,791 isolates (9.5%) that were identified to contain genes located within the CHASRI (supplementary data). Although only ~10% of the *Salmonella* isolates were CHASRI + , the future levels should be monitored to evaluate any impacts from agriculture practices. There were 30,374 (49.2%) isolates defined as clinical and 26,181 (42.4%) defined as environmental/other. After removing isolates with unknown sources or missing serotypes, the final data set was reduced to 52,177 isolates.

**Fig 1:**
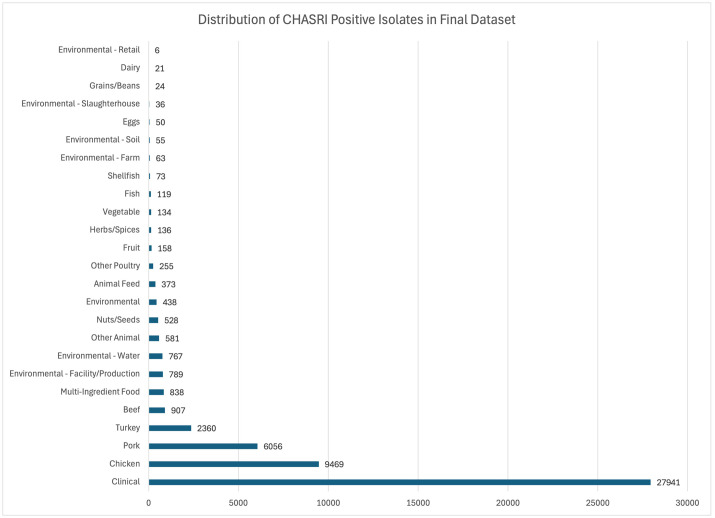
Source distribution of CHASRI-positive isolates by modified IFSAC category. Distribution of the 52,177 isolates containing the CHASRI across the 25 standardized source categories.

The distribution of isolates from the modified IFSAC source categories are illustrated in Fig 1. A total of 19,628 (81%) isolates represented livestock and other animal sources with the majority derived from chicken (39%) and pork (25%). The USDA Food Safety Inspection Service contributes many isolates to NCBI PD from the intensive poultry sampling program (https://www.fsis.usda.gov/science-data/sampling-program). The elevated number of CHASRI+ isolates derived from chicken and swine sources correlated with previously studies and further highlights the connection between copper supplementation and the selective pressure for bacteria to acquire heavy metal tolerance genes [11,20,21]. CHASRI isolates from food/environmental sources totaled 4,608, with the majority from multi-ingredient food (18.2%) followed by environmental – facility/production (17.1%) and environmental – water (16.6%). Additionally, sources that are typically considered low-moisture foods such as nuts/seeds, herbs/spices, grains/beans, and animal feed made up 23% of the food/environmental isolates. However, there is the possibility of potential bias from over-representation of CHASRI-positive isolates from low-moisture foods due to increased testing during outbreak investigations where these types of products (nuts, peanut butter, flour) have been implicated.

The date of collection of the isolates ranged from 1972–2024. Many of the isolates were collected in the United States, mostly due to the robust sequencing networks of FDA (GenomeTrakr), USDA, and CDC (PulseNet); however, CHASRI + isolates also originated from the Americas, Europe, Asia, and Oceania.

**Fig 2:**
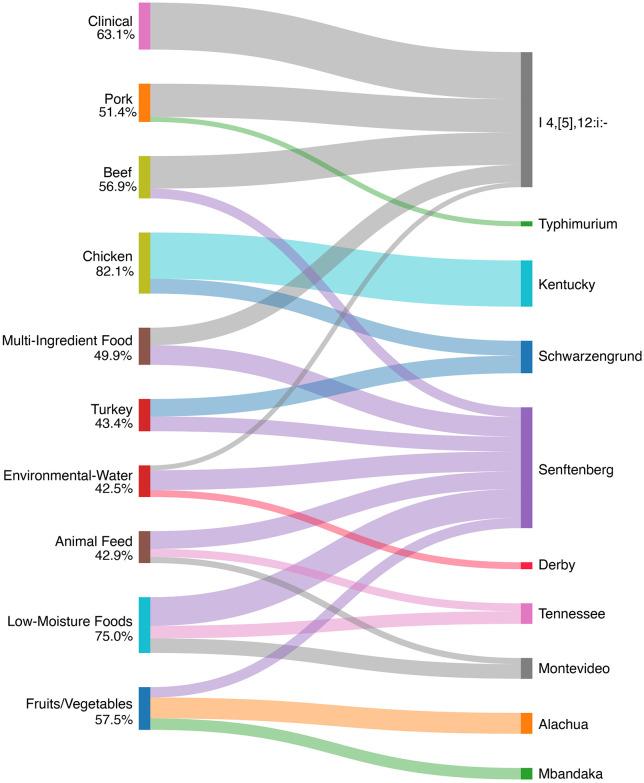
Distribution of predominate *Salmonella* serovars across selected isolation sources for CHASRI-positive isolates. Sankey Diagram illustrating the relationship between isolation sources (left) and the most prevalent serovars (right) identified in the dataset. Line thickness is proportional to the percentage of isolates within each source category that were identified as the corresponding serovar. The percentage is the total for those given serovars within the sources.

Fig 2 highlights the top serovars for a subset of isolation sources. The line thickness corresponds to the percentage of isolates that were typed as that serovar. The majority of the clinical isolates were typed serovar I 4, [5],12:i:- (63%), which have been previously linked to consumption of contaminated swine and poultry [22,23]. I 4,5 [12]:i:- was the predominant serovar associated with the pork-derived products (44.7%). Serovars Kentucky and Schwarzengrund, which are considered an MPPST (most prevalent poultry-associated *Salmonella* serotypes), made up 82% of the chicken isolates [24]. Low moisture foods were predominantly associated with Senftenberg (38.5%), Montevideo (19.6%), and Tennessee (16.9%). Interestingly, outbreaks associated with nuts/seeds and spices from these serovars had isolates that contained the CHASRI [10,25,26]. The complete breakdown of serovar distribution for each of the 25 source categories is included in the supplementary materials.

The CHASRI has been noted to be present in particular *Salmonella* serovars (I 4 [5],12:i-) and in particular sequence types (ST316, ST34) [7,11,27], but little is known about other serovars that may also contain the island. MLST analysis of our dataset identified that the Typhimurium isolates that had the CHASRI were associated with ST34 and ST19, which were also associated with I 4, [5],12:i:-. Most of the other serotypes from the dataset were typed as a single sequence type. Based on the top 15 serovars in our dataset, the overall incidence rate was determined for the top 15 serovars in this study as well as the top serovars found in the Pathogen Detection database (Table 1). Although I 4, [5],12:i:- accounted for the serovar with the highest number of isolates in our study, four other serovars from the dataset had a higher incidence rate (Worthington, Senftenberg, Alachua, Schwarzengrund). Interestingly, a previous study of the phylogeny of *Salmonella* based on whole genome sequencing and patterns of acquired genomic elements, grouped Senftenberg and Alachua in the same clade (A1) [28]. Identifying these relationships can provide insights into the acquisition of the CHASRI in different serovars. Perhaps serovars found in the same clade acquired the CHASRI from a common ancestor, whereas serovars in different clades may have acquired the CHASRI due to environmental influences. Increased surveillance and sequencing of *Salmonella* found in all environments has the potential to increase the numbers of different serovars. This would provide further evidence of the overall phylogeny of *Salmonella* and the movement of genomic islands.

**Table 1:** CHASRI incidence rates across *Salmonella* serovars in NCBI Pathogen Detection database. Comparison of the top 16 serovars from the current study and the five most abundant serovars overall in the NCBI Pathogen Detection database (marked with asterisks). Incidence rates are calculated from the complete NCBI database.

Rank in this dataset	Serovars	Total Number of Isolates containing the CHASRI	Total Number of Isolates in Pathogen Detection	Incidence Rate % (95% CI)
1	I 4,[5],12:i:-*	22,870	30,807	74.2 (73.5–75.0)
2	Kentucky	6,785	16,914	40.1 (39.0–41.3)
3	Senftenberg	3,985	4,330	92.0 (90.7–93.2)
4	Schwarzengrund	3,547	4,624	76.7 (74.8–78.6)
5	Typhimurium*	4,513	75,056	6.0 (5.8–6.3)
6	Montevideo	1,387	12,102	11.5 (10.6–12.4)
7	Heidelberg	1,310	8,202	16.0 (14.8–17.2)
8	Rissen	1,059	1,539	68.8 (65.2–72.3)
9	Worthington	842	894	94.2 (91.4–96.3)
10	Derby	1,719	4,828	35.6 (33.5–37.7)
11	Mbandaka	934	5,201	18.0 (16.4–19.6)
12	Alachua	674	781	86.3 (82.2–90.0)
13	Ohio	681	1,320	51.6 (47.4–55.8)
14	Tennessee	650	953	68.2 (63.5–72.7)
15	Panama	631	4,097	15.4 (13.7–17.1)
16	Agona	710	9,120	7.8 (7.0–8.7)
21	Infantis*	565	32,613	1.7 (1.5–2.0)
47	Enteritidis*	93	111,952	0.1 (0.1–0.1)
53	Newport*	88	44,708	0.2 (0.1–0.3)

### Analysis of reference genomes of isolates for further characterization of the CHASRI

**Fig 3:**
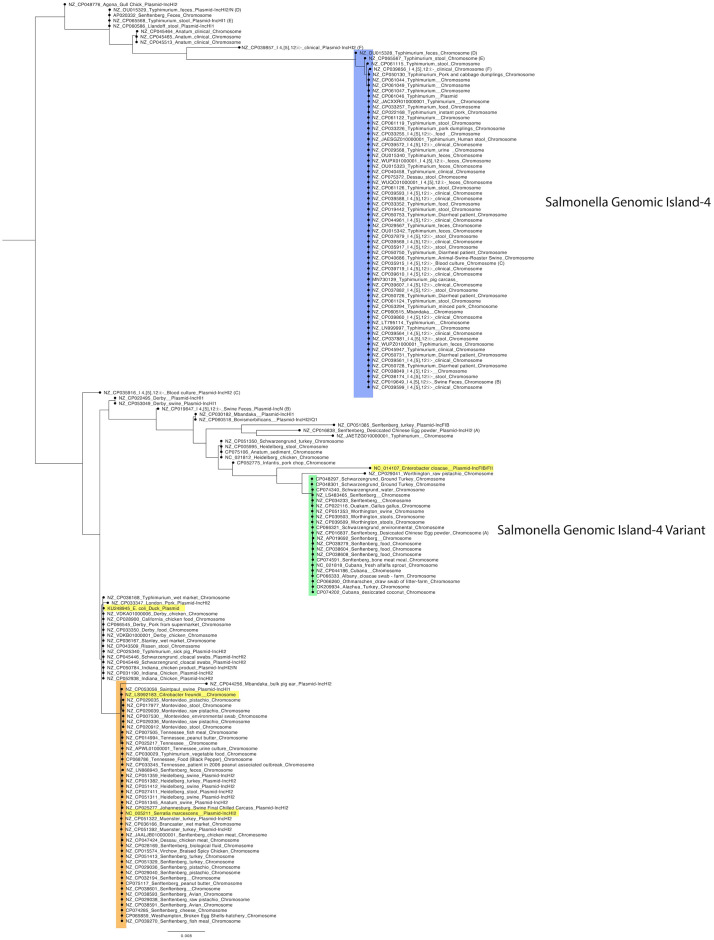
Phylogenetic analysis of the CHASRI sequences reveals multiple acquisition pathways in *Salmonella enterica.* Maximum likelihood tree constructed from the 171 CHASRI nucleotide sequences (35,204 base pair alignment) extracted from *Salmonella* reference genomes and plasmids, with four non-*Salmonella* sequences included for comparison (highlighted in yellow). Tree nodes are color-coded by acquisition type: blue indicates isolates containing the *Salmonella* Genomic Island-4, green indicates SGI-4 variant, and orange indicates an independent mobile genomic element. Matching letters (A-F) identify six isolates where CHASRI was found on both chromosome and plasmid.

Reference genomes were used to determine how the CHASRI is integrated into *Salmonella* genomes and to identify any accessory genes that may be associated with the CHASRI genomic mobile element. A total of 171 nucleotide sequences of the CHASRI which were extracted from reference genomes and plasmids were aligned and used for the maximum likelihood tree in Fig 3. The nucleotide length of the CHASRI region alignment was 35,204 base pairs. The metadata for the isolates can be found in supplementary materials.

The CHASRI was located on the chromosome in 135 isolates and on a plasmid in 36 isolates; however, the location of the CHASRI did not correlate with any phylogenetic characteristics. The different plasmid types containing the CHASRI in *Salmonella* were typed as IncHI2 plasmids (n = 23/33), IncHI1 (n = 6/33), IncN (n = 1/33), and IncFIB (n = 1/33). The plasmid types were included in the tree labels.

The 167 CHASRI sequences derived from *Salmonella* are from 32 different serovars, the most abundant being Typhimurium (25.7%), I 4, [5],12:i:- (16.2%), and Senftenberg (15.6%).

There was a total of 63 isolates that contained SGI-4 (nodes marked in blue) and 22 isolates that contained SGI-4 variants (nodes marked in green). There were also six instances where the CHASRI was found on both the chromosome and plasmid in the same isolate, which is denoted by the matching paired letters (A-F) on the tree. Interestingly, none of these paired CHASRIs had identical sequences. Only two paired isolates TW-Stm6 (B) and S61394 (C) had intact CHASRIs located on both chromosome and plasmid but with a pairwise identity of 93.5%. Three of these six isolates have the CHASRI operon intact on the chromosome, however the CHASRIs found on a plasmid (NZ_OU015329 (D), NZ_CP065568 (E), and NZ_CP039857 (F)) have truncated or missing *pcoD, pcoR and pcoS* genes*.* The isolate 775W ATCC43845 (A) has an insertion of a transposase in the CHASRI located in the chromosome (NZ_CP016837) but an intact CHASRI on the plasmid (NZ_CP016838).

The orange highlighted area on the tree is comprised of the CHASRI sequences from 42 *Salmonella* isolates as well as the CHASRI from the *S. marcescens* and the *C. fruendii* isolates. The CHASRI sequences from this group were similar to the CHASRI from *S. marcescens* (NC_005211), which was denoted as “P3 CHASRI” in the publication by Staehlin et al (2016) and, based on their molecular clock study, was determined to be a more recent introduction of the CHASRI [4]. In our analysis, this CHASRI sequence was identified in strains associated with previous outbreaks of salmonellosis in low moisture foods including pistachios (Montevideo, Senftenberg, Worthington), spices (Montevideo), and peanut butter (Tennessee) [26,29–31].

The isolates highlighted in blue on the tree contain the SGI-4, and the CHASRI sequence for these isolates is very similar. The 63 sequences that contained the SGI-4 cassette were predominately serovar Typhimurium or I 4, [5],12:i:-; however, there are single isolates of serovars Dessau and Mbandaka present. There were eight isolates that were below the BLAST cutoff for 85% coverage, but these isolates were inspected visually and did contain the SGI-4. An insertion of the gene *aph(3’)-II* occurred in the region between DUF2933 and *pcoG* in four of the isolates (1 plasmid:NZ_CP061046, 3 chromosome:NZ_CP061044, NZ_CP06147, NZ_CP06149). Excluding those sequences with the AMR insertion, the sequences had a median SNP distance of 0, where the largest difference in the sequences was found in the *pcoB* gene of NZ_OU15328. The *pcoB* gene encodes an outer membrane protein that may inhibit the uptake of copper; it is unknown if these changes may alter the function of this protein [32].

**Fig 4:**
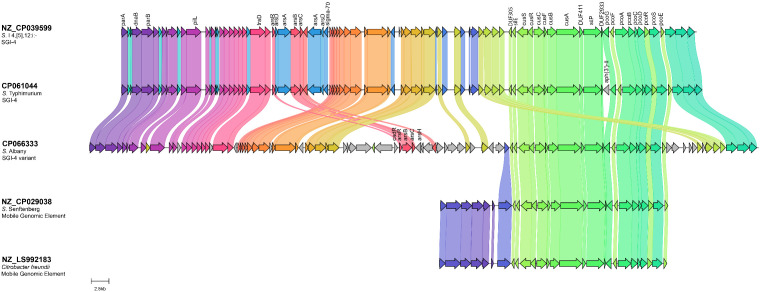
Genome Comparison of the different versions of the CHASRI found in the closed genomes from our analysis produced with Clinker software. Shared gene segments between the strains are denoted by fill color.

The SGI-4 variant is found in those isolates highlighted in green on the tree. The CHASRI sequences from SGI-4 (MN730129.1) and SGI-4 variant (OK209934.1) are not closely related (64.6% pairwise similarity) The main difference between the SGI-4 and the variant is found within the arsenic operon, as shown in fig 4. The SGI-4 variant contains a different combination of arsenical genes than the typical SGI-4. Interestingly, the SGI-4 and the variant CHASRI sequences are on separate nodes of the tree. There is a median of 1,008 differences between the CHASRI sequences from these two islands, indicating the SGI-4 and its variant has been evolving separately for a substantial amount of time. The CHASRI sequence for the 21 isolates that contained the SGI-4 variant was conserved with a median 0 SNPs (range 0–5), suggesting that the variant has likely stabilized in a separate population or environment, possibly due to strong selective pressures favoring its current form. The abundance of SNP discrepancies between SGI-4 and its variant, in addition to the different *ars* operon, implies that the SGI-4 variant emerged through horizontal gene transfer or a unique recombination event, leading to a distinct evolutionary path separate from the original SGI-4.

## Conclusions

*S. enterica* has been isolated from a large variety of sources and can survive in many different environments. The CHASRI is a genomic island that has become integrated in *Salmonella,* possibly due to outside pressures from extensive copper usage. This study shows that the CHASRI is prevalent in other serovars, for example Senftenberg, Tennessee, and Montevideo, beyond the previously reported serovar Typhimurium. The CHASRI is also found in sources not typically associated with poultry or pork such as low moisture foods, fruits and vegetables.

The phylogenetic analysis determines that the CHASRI is acquired in *Salmonella* through multiple pathways based on the presence of SGI-4, SGI-4 variant, and the mobile genetic element (MGE). There is evidence of the CHASRI in isolates derived from low-moisture sources that are not associated with SGI-4 or the variant. Future studies will be conducted to investigate if the CHASRI gives any advantage to *Salmonella* in a low moisture environment as well as protection during a response to an external stress. This analysis also identifies that the SGI-4 and the variant are not similar, both in nucleotide sequence or gene content, implicating that the SGI-4 variant evolved separately, acquiring different genetic features and adapting to different environments or selective pressures, likely through independent genetic events.

This study establishes a baseline for the prevalence of the CHASRI in *Salmonella enterica* using the public NCBI database. Monitoring the uptake of this island is important to evaluate in conjunction with increased use of copper as an organic pesticide, and nutrient supplement in crops and livestock. Future studies should test whether phenotypic differences among the variants of the CHASRI play a role in bacterial survival and persistence.

## Supporting information

S1 File Supplementary Data. (XLSX)
